# Case Report: complete response to preoperative ipilimumab and nivolumab combination therapy in advanced papillary renal cell carcinoma with inferior vena cava tumor thrombus and multiple metastases

**DOI:** 10.3389/fonc.2025.1547387

**Published:** 2025-08-26

**Authors:** Chika Nagahisa, Kazuhiko Yoshida, Shuhei Nozaki, Shinsuke Mizoguchi, Hironori Fukuda, Yuki Kobari, Junpei Iizuka, Yoji Nagashima, Hideki Ishida, Toshio Takagi

**Affiliations:** ^1^ Department of Urology, Tokyo Women’s Medical University, Tokyo, Japan; ^2^ Department of Surgical Pathology, Tokyo Women’s Medical University, Tokyo, Japan

**Keywords:** papillary renal cell carcinoma, inferior vena cava tumor thrombus, open nephrectomy, ipilimumab, nivolumab

## Abstract

**Introduction:**

Combination therapy comprising ipilimumab and nivolumab is a safe and effective treatment for advanced renal cell carcinoma (RCC). However, its use in papillary RCC (pRCC) with an inferior vena cava (IVC) tumor thrombus extending to the right atrium has not been reported. Herein, we describe a case of pRCC with an IVC tumor thrombus extending to the right atrium and multiple metastases, which was effectively treated with combination therapy comprising ipilimumab and nivolumab.

**Methods:**

A 74-year-old man was diagnosed with pRCC based on a computed tomography-guided biopsy of a left renal tumor. The tumor extended to the right atrium and was accompanied by metastases to the pancreas, retroperitoneum, lymph nodes, bones, and lungs (cT3cN2M1). Based on the International Metastatic Database Consortium risk classification, the case was categorized as poor risk. Therefore, combination therapy comprising ipilimumab and nivolumab was initiated.

**Results:**

Nine months after initiating treatment, all metastases had disappeared, enabling open cytoreductive nephrectomy that had been deferred. Histopathological analysis revealed necrosis in most of the tumor tissue, with no viable cells in the primary tumor and IVC tumor thrombus. At 24 months postoperatively, the patient remained recurrence-free.

**Discussion:**

This case highlights the potential efficacy of preoperative combination therapy with ipilimumab and nivolumab in advanced pRCC with IVC tumor thrombus and distant metastases. The treatment achieved a complete response and allowed successful surgical resection, suggesting its potential as a viable option in similar cases.

## 1 Introduction

Combination therapies involving immune checkpoint inhibitors (ICIs) have markedly improved the treatment of clear cell (cc) renal cell carcinoma (RCC). However, data on non-cc (ncc) RCC remain limited and are primarily derived from small-scale or retrospective trials (1). Some nccRCC subtypes exhibit more aggressive behavior than ccRCC in RCC with thrombosis (2). Few systemic treatment trials have included patients with metastatic nccRCC, and their reported efficacy has been modest compared to that of treatments for ccRCC (3). While the combination of nivolumab and ipilimumab is the standard treatment for metastatic ccRCC, its effectiveness against nccRCC has not been fully examined (4). The role of combination ICIs in treating inferior vena cava (IVC) tumor thrombi remains unclear. To date, there are no reports detailing the use of ipilimumab and nivolumab combination therapy specifically for papillary RCC (pRCC) with IVC tumor thrombi extending to the right atrium. Here, we report a case of advanced pRCC with an IVC tumor thrombus that was preoperatively treated with ipilimumab and nivolumab combination therapy (5). Furthermore, this is the first reported case in which CR was achieved in a cT3c, stage 4 case, with no recurrence for as long as 24 months.

## 2 Case report

**Figure 1:**
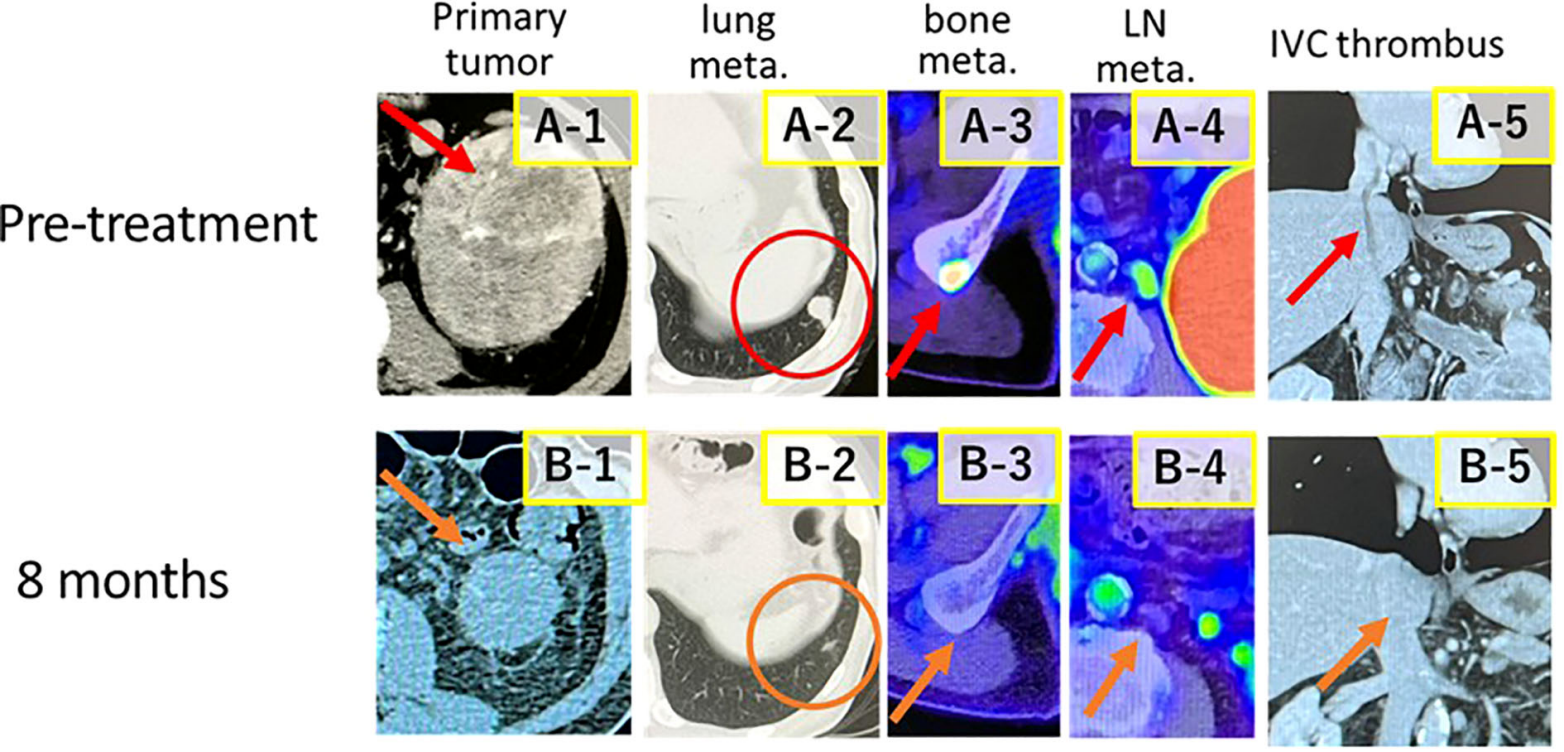
Computed tomography and positron emission tomography-computed tomography images (before treatment, red arrows; after treatment, orange arrows). Images of the primary tumor, lung metastases, bone metastases, lymph node metastases, and inferior vena cava (IVC) tumor thrombus. (A-1, A-2, A-3, A-4, and A-5). Images of the primary tumor and IVC tumor thrombi 8 months after combination treatment with ipilimumab and nivolumab are shown (B-1, B-2, B-3, B-4, and B-5).

A 74-year-old man presented with gross hematuria and a left renal mass, leading to a referral to our department for further evaluation and treatment. Radiological examinations revealed an enhanced tumor in the left kidney with a maximum diameter of 103 mm, an IVC tumor thrombus extending to the right atrium (level 4 according to the Neves-Zincke classification system) (5), metastases to the pancreas, retroperitoneum, lymph nodes, and bones, as well as multiple pulmonary metastases (**Figure 1A**).

Results of the computed tomography-guided percutaneous kidney tumor biopsy indicated a pathological diagnosis of pRCC (cT3cN2M1, stage IV). Based on the International Metastatic RCC Database Consortium risk classification, this case was categorized as poor due to the following factors: elevated corrected calcium levels, a duration of less than 1 year between initial diagnosis and treatment initiation, anemia, and neutrophilia (6). Because the patient had cT3cN2M1 status and initially chose to continue treatment with pharmacotherapy rather than surgery, IOIO was selected instead of IOTKI.

The patient received four courses of combination therapy comprising ipilimumab and nivolumab. However, 5 months after treatment initiation, type 1 diabetes developed, necessitating temporary discontinuation of nivolumab treatment. At the onset of diabetes, the patient’s blood glucose level was 442 mg/dL, and the urinary ketone level was 1+. Therefore, diabetic ketoacidosis was diagnosed, and insulin therapy was initiated after referral to the endocrinology department. Nivolumab treatment was resumed 6 weeks thereafter.

During treatment, the tumor progressively shrank. At 8 months after treatment initiation, the primary tumor’s diameter had decreased to approximately 40% of its original size, the IVC tumor thrombus level had reduced from level 4 to level 1, and pulmonary metastases were no longer detectable on computed tomography (**Figure 1B**). At 10 months after treatment initiation, the patient underwent open left nephrectomy as deferred cytoreductive nephrectomy (CN).

The operative time was 271 min, with blood loss totaling 1,170 mL. Intraoperatively, dissection was challenging due to adhesions around the renal tumor and pancreatic metastases. The surgery was completed without perioperative complications or the need for intraoperative blood transfusion. However, a 560-mL red blood cell transfusion was administered on postoperative day 1. The patient recovered well and was discharged on postoperative day 8.

**Figure 2:**
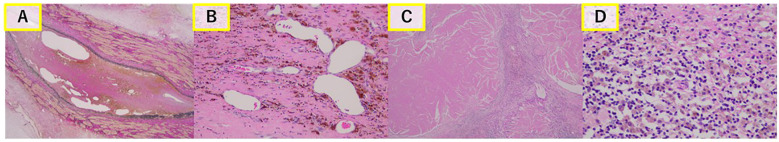
Histopathological findings. Most cells in the inferior vena cava (IVC) tumor thrombus **(A)** and primary tumor **(C)** are necrotic. No viable cells are observed in the primary tumor **(B)** or IVC tumor thrombus **(D)**.

Histopathological examination confirmed pRCC with a negative surgical margin. Most of the tumor tissue was necrotic, with no viable cells detected in the primary tumor or IVC tumor thrombus (**Figure 2**). Postoperative imaging demonstrated a complete response, and nivolumab treatment was discontinued. At 24 months postoperatively, the patient remained recurrence-free.

## 3 Discussion

The role and timing of offering nephrectomy in the contemporary immunotherapy era remain largely undefined, and until now, the feasibility and safety of performing nephrectomy after prior receipt of ICI have not been studied systematically. Nirmish Singla et al. reported that nephrectomy following ICI for RCC appears to be both safe and technically feasible with favorable surgical outcomes and pathologic response (7).

We report the case of a patient with advanced pRCC, an IVC tumor thrombus extending to the right atrium, and multiple metastases who was preoperatively treated with combination therapy comprising ipilimumab and nivolumab. This therapy allowed for open nephrectomy and IVC thrombectomy. A complete response was achieved, and computed tomography imaging following deferred cytoreductive nephrectomy showed the absence of metastases.

pRCC is the second most common RCC subtype, accounting for 10–15% of all RCC cases (8). Patients with pRCC that extends to the IVC have significantly shorter survival than that of patients with ccRCC. Few studies have reported tumor thrombus extension in pRCC and have associated it with worse cancer-specific outcomes compared with ccRCC (9). The median survival for patients with pRCC and lymph node or distant metastases is only 5.2 months (10). We previously reported that patients with pRCC extending to the IVC may not be suitable candidates for extensive surgery when lymph node or distant metastases are present (10). However, radical surgery remains the only option that may allow long-term survival of patients with RCC and an IVC tumor thrombus. For patients presenting with an IVC tumor thrombus and metastases, the longest survival is achieved through cytoreductive surgery combined with immunotherapy (11). Preoperative treatment can reduce IVC tumor thrombi, thus decreasing the invasiveness of surgery. In these cases, radiation therapy is considered if the tumor does not significantly increase or change after drug therapy. Valentina Zagardo et al. reported on the potential use of radiation therapy as a safe and well-tolerated treatment for inoperable or metastatic patients with inferior vena cava tumor thrombus (12).

Based on the results of the CheckMate 214 trial, a multicenter, randomized, open-label, phase III clinical trial, combination therapy comprising ipilimumab and nivolumab was approved for intermediate-risk and high-risk metastatic RCC worldwide in 2018 and in Japan in August 2018 (13). However, the benefits and clinical efficacy of immune checkpoint inhibitors in nccRCC remain uncertain and have yet to be fully defined (8, 14). The results of the CheckMate 920 trial, a multicenter, randomized, open-label, phase IIIb/IV clinical trial evaluating the safety and efficacy of nivolumab plus ipilimumab for patients with advanced nccRCC, were reported in 2022 (15). Treatment-related complications observed during the trial included diarrhea/colitis (7.7%), rash (5.8%), nephritis and renal dysfunction (3.8%), hepatitis (1.9%), adrenal insufficiency (1.9%), and hypophysitis (1.9%) (15), all of which were grade 3 or 4 immune-mediated adverse events (AEs). No grade 5 immune-mediated AEs were reported (15). Additionally, Bimbatti et al. reported a progression-free survival of 12.7 months and an overall response rate of 42.4% for patients with nccRCC treated with first-line combination immunotherapy.

In the present case, combination therapy comprising ipilimumab and nivolumab was chosen based on the patient’s condition. This treatment eliminated metastases to the lungs, bones, and lymph nodes without surgical intervention. Furthermore, this treatment resulted in significant shrinkage of the primary tumor and IVC tumor thrombus extending to the right atrium, enabling open nephrectomy. Furthermore, the multiple metastases achieved a radiological complete response, and no treatment was required postoperatively. The patient remained recurrence-free during the 24-month follow-up period. To our knowledge, this is the first case report of a patient with cT3c, stage 4 cancer who achieved a radiological complete response without long-term recurrence.

In this case, type 1 diabetes developed as a treatment-related side effect 5 months after therapy initiation. Similar to classic type 1 diabetes, ICI-associated diabetes mellitus is likely caused by immune-mediated destruction of pancreatic beta cells. Symptoms typically present within 6 months of ICI initiation, although their onset is unpredictable and may occur at any point during or even after therapy. Diabetes mellitus can be life-threatening in patients presenting with diabetic ketoacidosis (16).

In this case, nivolumab treatment was temporarily discontinued but resumed 2 months after initiating insulin therapy. Tumor shrinkage continued following the resumption of nivolumab treatment. Based on the patient’s clinical course, we believe it is prudent to initiate appropriate management of AEs while continuing therapy whenever possible.

Recent treatment recommendations include immuno-oncology-tyrosine kinase inhibitor (TKI) combinations and TKI monotherapy. However, ipilimumab and nivolumab therapy for nccRCC is not currently recommended in the guidelines (17). In our patient with pRCC and distant metastases, combination therapy with ipilimumab and nivolumab significantly reduced the tumor size, allowing cytoreductive nephrectomy, which had been deferred. A pathological examination revealed the absence of viable cells in both the primary tumor and the IVC tumor thrombus, and the patient required no further treatment for 24 months.

In conclusion, preoperative combination therapy comprising ipilimumab and nivolumab eliminated the primary tumor and multiple metastases, enabling open nephrectomy in this patient with stage IV pRCC, an IVC tumor thrombus extending to the right atrium, and multiple metastases at presentation and he has remained free of long-term recurrence. These findings suggest that combination therapy comprising ipilimumab and nivolumab may be effective for pRCC.

## Data Availability

The original contributions presented in the study are included in the article/supplementary material. Further inquiries can be directed to the corresponding author.
